# Quinazoline derivative QPB-15e stabilizes the *c-myc* promoter G-quadruplex and inhibits tumor growth *in vivo*

**DOI:** 10.18632/oncotarget.9088

**Published:** 2016-04-28

**Authors:** Zeng Li, Chen Liu, Cheng Huang, Xiaoming Meng, Lei Zhang, Jinhui He, Jun Li

**Affiliations:** ^1^ School of Pharmacy, Anhui Medical University, Hefei 230032, China; ^2^ School of Pharmaceutical Sciences, Sun Yat-sen University, Guangzhou University City, Guangzhou 510006, China

**Keywords:** c-myc, G-quadruplex, quinazoline derivative, QPB-15e, anti-tumor

## Abstract

The ribozyme-sensitive element NHE-III1 in the P1 promoter region of the important proto-oncogene *c-myc* contains many guanine (G)-rich sequences. Induction and stabilization of the G-quadruplex formed by NHE-III1 can downregulate *c-myc* expression. In the present study, we found that QPB-15e, a quinazoline derivative designed and synthesized by our laboratory, binds to and stabilizes the *c-myc* G-quadruplex *in vitro*, thereby inhibiting double-stranded DNA replication, downregulating *c-myc* gene expression and arresting cancer cell proliferation. PCR termination experiments showed that QPB-15e blocked double-stranded DNA replication by inducing or stabilizing the *c-myc* G-quadruplex. FRET-melting further confirmed that QPB-15e improved the stability of the G-quadruplex, and CD spectroscopy indicated that the compound interacted directly with the G-rich sequence. In competitive dialysis experiments, QPB-15e bound preferentially to quadruplex DNA in various structures, especially the G-quadruplex within the *c-myc* promoter region. Moreover, QPB-15e reduced the weights and volumes of tumors transplanted into nude mice. These findings strongly suggest that QPB-15e is a *c-myc* G-quadruplex ligand with anti-tumor properties, and may be efficacious for treating cancer in humans.

## INTRODUCTION

The human *c-myc* oncogene is a pluripotent cytokine and a key regulator of many physiological processes, including cell-cycle control, protein synthesis, apoptosis and cell adhesion [1]. Abnormal *c-myc* expression, often as a result of direct gene alteration, is associated with tumorigenesis and sustained tumor growth [2–4]. Many factors may cause *c-myc* gene expression disorders, such as oncogene activation or tumor suppressor loss or inactivation [5]. According to recent studies, about 20% of human tumors can be associated with *c-myc* overexpression. Thus, anti-*c-myc* therapies have become a focus in the field of cancer therapeutics, and include antibodies [6] and small-molecule inhibitors [7–8].

G-quadruplex DNA is a type of secondary DNA structure that originates from the assembly of four guanine (G)-rich DNA strands together as a G-quartet [9]. G-quadruplex DNA was discovered in eukaryotic telomere and oncogene promoters through a G-quadruplex-specific antibody and was shown to be a potential small molecule target [10–11].*c-myc* transcription is primarily regulated by a 27-base G-rich sequence in the NHE III1 (nuclease hypersensitivity element III1) region [12–13]. This sequence is located upstream (−142 to −115 bp) of the P1 promoter in the human *c-myc* oncogene and controls 85–90% of *c-myc* transcription [14]. This tract can form specific G-quadruplex structures enhanced by G-quadruplex-interactive ligands, leading to *c-myc* downregulation in human tumor cells [15–16]. Small molecules that can selectively bind to and stabilize the *c-myc* G-quadruplex are likely to be effective therapeutics for cancer treatment.

Recently, we synthesized quinazoline derivatives with an “imitative” tetracyclic aromatic system formed through intramolecular hydrogen bonding; we also demonstrated the derivatives' binding to telomeric G-quadruplex DNA [17–18]. These molecules significantly affected telomere function, inducing telomere shortening, telomerase inhibition, senescence proliferation and apoptosis. In particular, the quinazoline derivative QPB-15e, with a new benzene ring attached to the quinazoline derivative scaffold by an amide bond, showed the highest potential activity among reported quinazoline derivatives [18]. Some targeted G-quadruplex telomerase inhibitors stabilize the *c-myc* promoter region G-rich sequence to form a G-quadruplex. Properly regulated *c-myc* expression is important for tumor prevention and treatment [15–16]. In the present study, the interaction of QPB-15e with a G-rich sequence located upstream of the *c-myc* NHE III1 region was investigated, and the effects of this interaction on *c-myc* function *in vitro* and *in vivo* were determined. A typical transplanted hepatocellular carcinoma model in Balb/c-nude mice was used to investigate the tumor growth inhibiting effects of QPB-15e.

## RESULTS

### PCR stop and fluorescence resonance energy transfer (FRET)–melting assay

**Figure 1:**
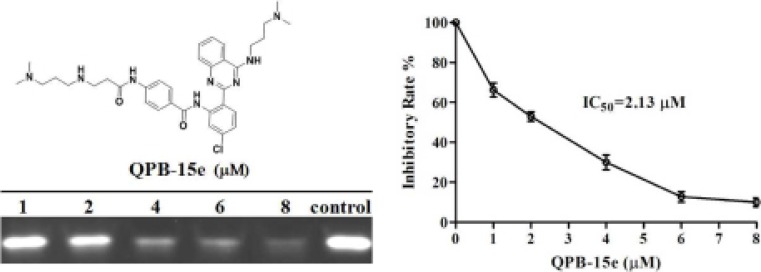
PCR stop assay with QPB-15e and *c-myc* Pu27 DNA

**Figure 2:**
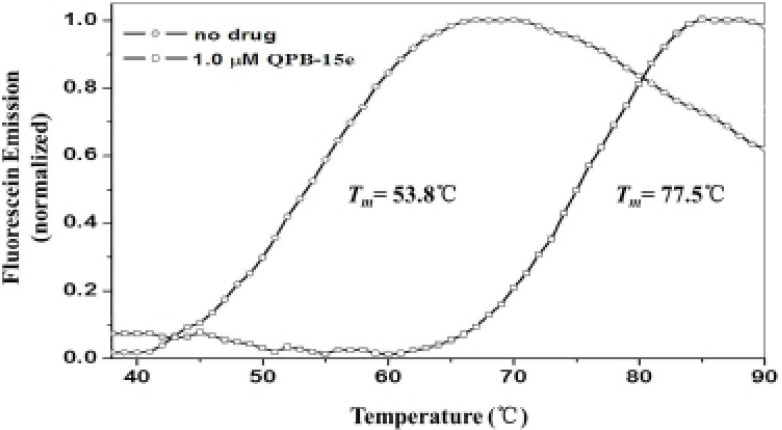
Pu18 melting curves in the absence and presence of QPB-15e

Inhibition of *c-myc* G-rich sequence expansion by QPB-15e *in vitro* was quantitatively studied using PCR stop assay. The *c-myc* gene single-stranded Pu27 (5′ - TGGGG AGGGTGGGGAGGGTGGGGAAGG - 3′) was combined with complementary strand Pu27rev (5′ - ATCGATCT CTTCTCGTCCTTCCCCA - 3′) by annealing to a 43 bp sequence to form a specific 43 bp double-stranded product by amplification [19]. Optical density of the double-stranded product bands decreased with increasing QPB-15e concentration, showing inhibition of double-stranded product synthesis (Figure 1). Ligand concentrations capable of decreasing PCR product synthesis by 50% (IC_50_) were calculated using optical densities from the gel images. QPB-15e (IC_50_ = 2.13 μM) inhibited DNA expansion *in vitro* by inducing and stabilizing the formation of the *c-myc* G-quadruplex, and this was further evaluated using the FRET–melting assay [20]. The oligomer Pu18 containing a fluorophore at the 5′ end and a fluorescence quencher at the 3′ end (FPu18T, 5′ - FAM–AGGGTGGGGAGGGTGGGG–TAMRA - 3′) was used in this experiment. The derivative could increase *c-myc* G-quadruplex stability with a Tm value of up to 77.5°C (Figure 2).

### Circular dichroism (CD) spectroscopy

**Figure 3:**
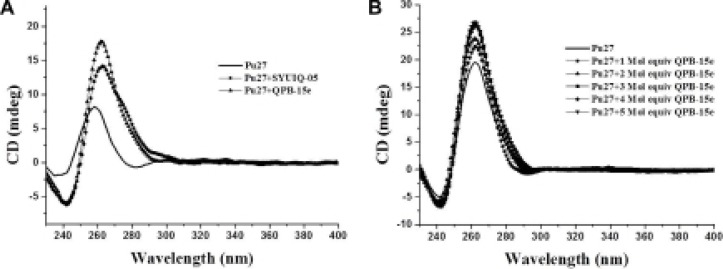
CD spectra of Pu27 DNA CD spectra of 5 μM Pu27 in the absence of cations in Tris–HCl buffer with SYUIQ-05 (control) or QPB-15e (**A**) CD titration spectra of Pu27 (5 μM) at increasing QPB-15e concentrations (arrows: 0–5 Mol equiv) in 10 mM Tris–HCl buffer, pH 7.2, with 100 mM KCl (**B**).

The binding of QPB-15e with the *c-myc* G-quadruplex was studied using CD spectroscopy [21]. SYUIQ-05 was selected as the positive control [22]. In the absence of salt, the CD spectrum of the randomized Pu27 oligonucleotide exhibited a negative band at 238 nm and a major positive band at 257 nm. After treatment with QPB-15e, the positive peak shifted to 260 nm and the negative peak at 240 nm increased, which indicated that QPB-15e could induce the *c-myc* G-rich sequence to form the parallel G-quadruplex structure (Figure 3A) [23]. In a K^+^ buffer solution, the positive peak gradually shifted from 256 nm to about 265 nm, and the negative band increased at around 240 nm with increasing QPB-15e concentration from 1 to 5 Mol equivalence (Figure 3B), thereby confirming the activation of the Pu27 sequence to form the parallel G-quadruplex.

### Competitive dialysis

We used competitive dialysis to investigate the selectivity of QPB-15e for different oligomers [24]. We selected 10 oligomers with different structures, including telomere sequence HT-7 with intermolecular positive parallel quadruplex structure; oncogene *c-myc* gene sequence Pu27 with positive parallel G-quadruplex structure; duplex DNA HTds (HTC21: HTG21, telomere sequence HTC21 and HTG21 were paired to form 21 base pairs); HTC21 d [(C_3_TAA)_3_C_3_], with i-motif DNA secondary structure, which was the complementary stand of HTG21 d [G_3_(T2AG_3_)_3_]; three-screw structure 2(dT21): dA21; telomere sequence HTG21mu d(GAG [T_2_AGAG]_3_), with single-stranded structure after multiple mutations; single-stranded dT21 with 21 pyrimidine bases; and single-stranded sequence dA21 with 21 purine bases.

**Figure 4:**
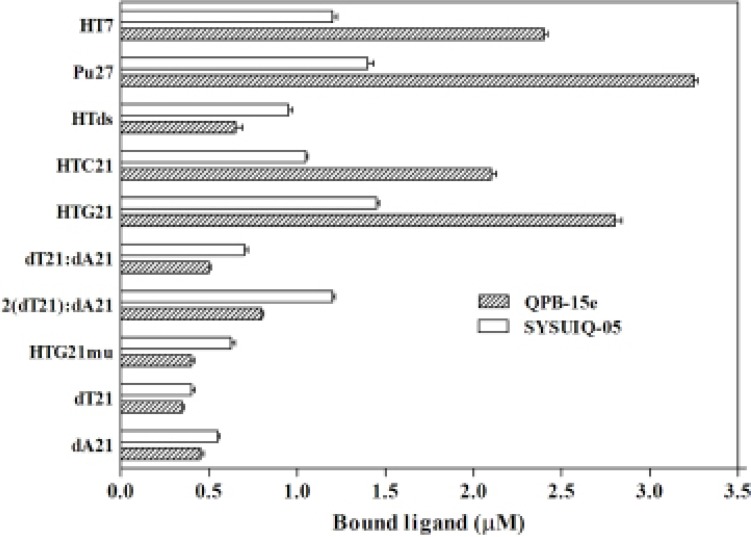
Results of the competition dialysis assay The amounts of QPB-15e and SYUIQ-05 bound to each structure are presented in a bar graph.

Compared with positive control, SYUIQ-05, binding of QPB-15e with single-, double-, and triplex-stranded DNAs (Figure 4) was reduced to varying degrees. However, binding of QPB-15e with quadruplex DNA, including intermolecular G-quadruplex HT-7 d(T_2_AG_3_T), intramolecular G-quadruplex Pu27, HTG21 and the secondary structure i-motif with C-rich sequences, significantly increased. In particular, Pu27 in the oncogene *c-myc* promoter region exhibited the strongest binding. These results suggested that QPB-15e selectively binds the *c-myc* G-quadruplex.

### Molecular modeling

**Figure 5:**
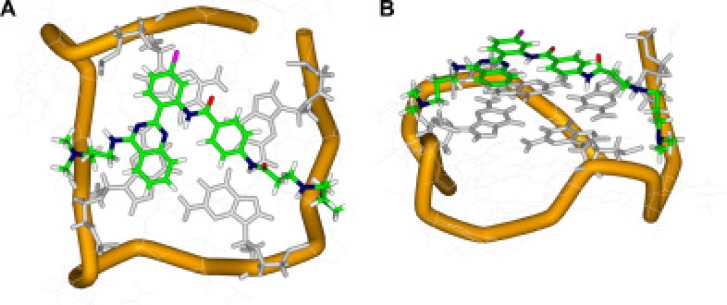
Top (A) and side (B) views of the docking structure of the complex between QPB-15e and *c-myc* G-quadruplex DNA

To elucidate the stability of the interaction between QPB-15e and the *c-myc* G-quadruplex and that of the derivative–quadruplex complex, we performed a molecular docking experiment. A sequence composed of Pu18 with *c-myc* G-rich sequence Pu27 and a quadruplex main body was used for large-molecule docking [25]. Docking results (Figure 5) revealed that QPB-15e tended to bind to the Pu18 5′ terminal G-quartet. The V-shaped aromatic parent nucleus and G-quartet exhibited good geometric matching, stacked on three guanines with its side chains in reversed grooves. The two side chains were extended to the groove from the tetrad plane, which also possessed a “V” shape. The fourth side chain was extended to the groove between base chains G12G13T14G15 and was bound with the loop comprising T14G15 by electrostatic and hydrogen-bond interactions. The side chain on the para position of the substituted benzene ring to the 2′ position of the phenyl group of the original core was extended to the groove between base chains G2G3G4T5G6. The terminal-end tertiary amine positive charge participated in electrostatic and hydrogen-bond interactions with a loop composed of the base chain G4T5. This finding was consistent with the thermal stability of the G-quadruplex in the presence of QPB-15e, which was attributed to stable π-π stacking interactions, hydrogen bonds and electrostatic interactions.

### QPB-15e binding to and stabilization of the *c-myc* G-quadruplex

The G-rich sequence in the *c-myc* promoter region could form a G-quadruplex with a small-molecule ligand. This interaction hinders binding of transcriptional activators, polymerases and other proteins, thereby inhibiting *c-myc* expression. We used reverse-transcription PCR (RT-PCR) and western blotting to show that QPB-15e induced/stabilized the G-quadruplex to downregulate *c-myc* expression.

**Figure 6:**
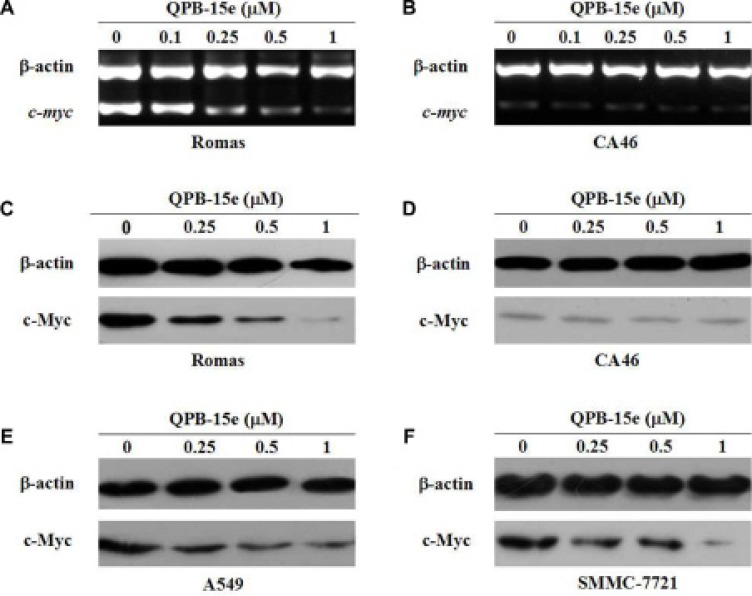
Effects of QPB-5e on *c-myc* transcription and translation RT-PCR (**A–B**) and western blotting (**C–D)** were conducted to determine *c-myc* mRNA and protein levels, respectively, in Ramos and CA46 cells treated with 0, 0.1, 0.25, 0.5, and 1 μM QPB-5e for 4 days. Western blotting to determine c-Myc protein levels in A549 and SMMC-7721 cells treated with 0, 0.25, 0.5, and 1 μM QPB-15e for 4 days (**E–F**).

We used a pair of cell models (lymphoma cell line CA46 and Ramos cell line) with differences in the *c-myc* promoter region sequence for experimental comparisons [26]. The CA46 cell line, which is deficient in the partial *c-myc* promoter region sequences, contained the NHE III1 element and cannot form the G-quadruplex structure, but only minimally expresses *c-myc*. The Ramos cell line contained the NHE III1 sequence. Total RNA from Ramos and CA46 cells was extracted and reverse transcribed to cDNA to investigate the effects of the derivatives on *c-myc* gene transcription. cDNA was then used as a template for the specific real-time PCR amplification of the *c-myc* sequence with β-actin as control. Results are shown in Figure 6A and 6B. After QPB-15e was added to Ramos cells, *c-myc* amplification products were dose-dependently reduced. When QPB-15e was added to CA46 cells, *c-myc* amplification products were expressed at low levels, similar to the negative control. To measure *c-myc* gene product translation, whole cell lysates were prepared, and c-Myc was detected by western blotting. c-Myc protein levels in Ramos cells decreased with increasing QPB-15e concentration (Figure 6C). c-Myc was expressed at a low level in CA46 cells, and levels did not change after QPB-15e was added (Figure 6D).

Under similar conditions, QPB-15e also downregulated c-Myc levels in the cancer cell lines, A549 and SMMC-7721 (Figure 6E–6F). Thus, we inferred that QPB-15e might induce/stabilize the *c-myc* promoter region to form a G-quadruplex, thereby decreasing *c-myc* expression.

### QPB-15e reduced tumor cell proliferation

QPB-15e achieved better *c-myc* inhibition in Ramos vs. C46 cells. We hypothesized that *c-myc* inhibition in the *c-myc*-overexpressing tumor cells would reduce tumor cell survival and proliferation.

**Figure 7:**
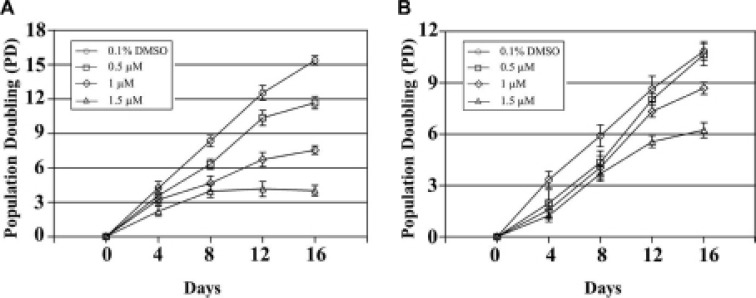
Long-term incubation of Ramos (A) and CA46 (B) cells in QPB-15e at subcytotoxic concentrations Experiments were performed in triplicate.

Based on MTT assay results (data not shown), we selected QPB-15e concentrations of 0.5, 1 and 1.5 μM for the long-term proliferation experiment (Figure 7). Ramos cell growth was inhibited on day four following treatment with 1.5 μM QPB-15e. Cells entered the proliferation platform stage on day eight. Growth was completely stopped on day 12 (Figure 7A). Cell proliferation was also inhibited after 1 μM QPB-15e was added on day 12. Compared with controls, time to logarithmic proliferation was significantly prolonged in the 0.5 μM experimental group (with the lowest concentration). The QPB-15e inhibitory effect was reduced in CA46 cells lacking the NHE III1 element as compared with Ramos cells (Figure 7B).

### QPB-15e inhibited SMMC-7721 liver cancer cell proliferation *in vivo*

**Figure 8:**
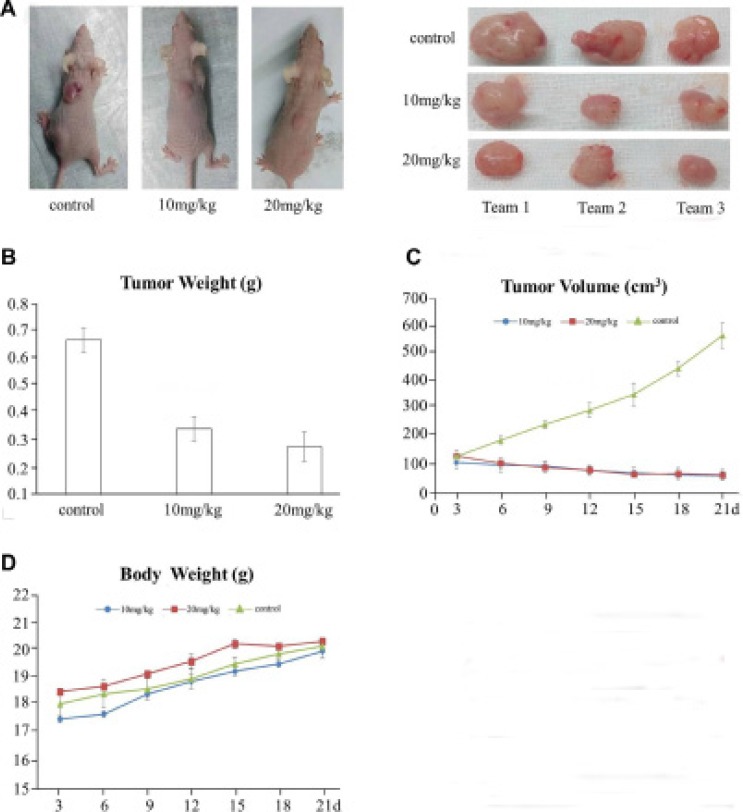
QPB-15e inhibits tumor growth in mouse xenografts SMMC-7721 cells (1 × 10^7^) were injected into 5-week-old female Balb/c nude mice. After tumors grew to 50–100 mm^3^, mice were given QPB-15e (10 or 20 mg/kg every day). Solid tumors in QPB-15e-treated mice were smaller than in untreated mice (**A–B**) QPB-15e inhibited tumor growth as measured by tumor volume (**C**) QPB-15e did not inhibit body weight increase compared with the control group (**D**).

We used the human liver cancer cell line, SMMC-7721, in a nude mouse transplanted tumor model. One of two QPB-15e concentrations (10 and 20 mg/kg) was injected intraperitoneally into mice daily, with saline as the negative control. Tumor inhibition rates were 49.3% and 58.2% with 10 and 20 mg/kg QPB-15e, respectively (*p* < 0.01) (Figure 8A–8B, Table 1). Tumor volumes and animal body weights were measured every three days following implantation. In the initial experimental stage, tumor volumes were not different between the experimental and control groups (Figure 8C). However, in the late experimental stage, tumor volumes in the experimental group were significantly lower as compared to controls. The average body weights of the experimental and control groups increased at the end of the drug administration, and no significant differences in weights were found among the three groups (Figure 8B). Thus, QPB-15e toxicity was relatively low.

**Table 1:** Effects of QPB- 15e on tumor weights and growth

Group	Tumor weight (g)	Inhibition rate
control	0.67 ± 0.04	
10 mg/lg	0.34 ± 0.04	49.3%
20 mg/kg	0.28 ± 0.05	58.2%

### QPB-15e inhibited *c-myc* expression in hepatocellular carcinoma xenografts

**Figure 9:**
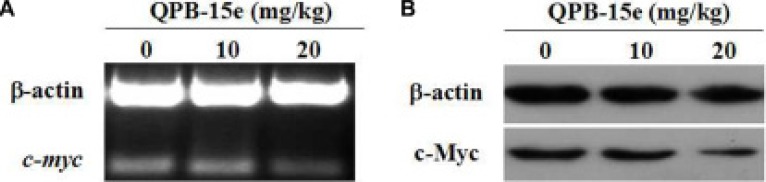
Effects of QPB-5e on *c-myc* expression in xenograft tumor tissue RT-PCR (**A**) and western blotting (**B**) were conducted to determine *c-myc* mRNA and protein levels, respectively.

*c-myc* mRNA and protein levels from tumor tissues were measured. Treatment with increasing concentrations of QPB-15e resulted in reduced *c-myc* expression compared with untreated tumors (Figure 9). Based on *in vitro* results, *c-myc* downregulation in treated tumors was likely due to QPB-15e interaction with the G-quadruplex structure in the *c-myc* promoter.

## DISCUSSION

The G-quadruplex DNA secondary structure is widely found in human gene promoters [27]. Development of small-molecule anticancer drugs interacting with the G-quadruplex has attracted extensive attention. Recent studies found that the ribozyme-sensitive element NHE III1 in the *c-myc* P1 promoter region contains the G-rich sequence, d [TGGGGAGGGTGGGGAG GGTGGGGAAGG], which binds transcriptional activation factors and DNA. When NHE III1 is single-stranded, the RNA polymerase catalytic reaction can be affected by various factors. After the G-quadruplex forms, the combination of various factors with the DNA single strand is inhibited. Formation and stabilization of the G-quadruplex could downregulate *c-myc* expression [12–14] and inhibit the growth and development of cancer cells to achieve an anticancer effect. A variety of *c-myc* G-quadruplex ligands are available, such as TMPyP4 [28], CX-3543 [29] and SYUIQ-5 [22]. The present study utilized the quinazoline derivative, QPB-15e, synthesized by our group, which can stabilize the telomeric G-quadruplex, thus regulating telomere function [18]. The G-quadruplex structure in the anti-apoptotic gene *c-myc* promoter region was regarded as an ideal target to investigate the ability of QPB-15e to suppress *c-myc* gene promoter activity.

The c-Myc protein plays important roles in multiple tumor growth processes [1]. PCR termination experiments showed that QPB-15e could block double-stranded DNA replication by inducing or stabilizing the formation of the *c-myc* G-quadruplex. FRET–melting further confirmed that QPB-15e could improve the stability of the *c-myc* G-quadruplex, and CD spectroscopy indicated that the compound could interact with the G-rich sequence directly. Under salt-free conditions, QPB-15e induced the *c-myc* gene sequence Pu27 to form a parallel G-quadruplex. This phenomenon also occurred in the K^+^ solution. Finally, competitive dialysis results showed that QPB-15e tended to bind with quadruplex DNA in various structures, especially the G-quadruplex in the *c-myc* promoter region. Along with molecular modeling, our *in vitro* results demonstrated that QPB-15e binds and stabilizes the *c-myc* G-quadruplex. QPB-15e might arrest cancer cell proliferation via multiple pathways, and the compound's specific mechanisms of action require further study.

We used a human liver cancer SMMC-7721 nude mouse transplanted tumor model to study the anti-tumor effects of QPB-15e *in vivo*. QPB-15e significantly inhibited SMMC-7721 cell tumor growth in mice and did not influence animal body weight. Thus, the compound exhibited low toxicity and good potential efficiency. Furthermore, QPB-15e inhibited *c-myc* expression in tumor tissues, possibly as a result of selective binding to the *c-myc* G-quadruplex. Further studies must be conducted to validate the anti-tumor QPB-15e mode of action.

In conclusion, our study confirmed that the quinazoline derivative, QPB-15e, designed and synthesized by our research group, reduced *c-myc* expression and exhibited anti-tumor effects *in vivo*. *In vitro*, we showed that QPB-15e selectively bound and stabilized the *c-myc* G-quadruplex and inhibited double-stranded DNA replication. Given these results, we will explore potential QPB-15e structural modifications for optimization of the compound as an effective human anti-cancer therapeutic.

## MATERIALS AND METHODS

### Materials

All chemicals were obtained from commercial sources unless otherwise specified. N-(5-chloro-2-(4-((3-(dimethylamino)propyl)amino)quinazolin-2-yl)phenyl)-4-(3-((3-(dimethylamino)propyl)amino)propanamido) benzamide (QPB-15e) was synthesized by our group, as previously reported [18]. The compound was identified by nuclear magnetic resonance (NMR) spectroscopy and high-resolution mass spectrometry. The purity of the compound was > 95%, as confirmed by high-performance liquid chromatography. The analysis results for QPB-15e are as follows: M.p. 127–128°C; ^1^H NMR (400 MHz, CDCl_3_) *d*: 14.58 (s,1H),11.16 (s,1H), 9.04 (d, *J* = 1.9 Hz,1H), 8.91 (s, 1H), 8.70 (d,*J* = 8.6 Hz, 1H), 8.11 (d, *J* = 8.5 Hz, 2H), 7.75–7.68 (m, 4H), 7.58 (d, *J* = 8.1 Hz,1H), 7.40 (t, J = 7.2 Hz,1H), 7.11 (dd, *J* = 8.6, 1.9 Hz, 1H), 3.82 (dd, *J* = 9.6, 5.2 Hz, 2H), 3.03 (t, *J* = 5.6 Hz, 2H), 2.80 (t, *J* = 6.6 Hz, 2H), 2.61 (t, *J* = 5.2, 2H), 2.56 (t, *J* = 5.6 Hz, 2H), 2.43–2.37 (m, 8H), 2.23 (s, 6H), 1.91–1.86 (m, 2H), 1.81–1.74 (m, 2H); ^13^C NMR (101 MHz, CDCl_3_) *δ*: 171.39, 166.00, 160.73, 159.15, 148.08, 141.93, 141.49, 136.79, 132.79, 131.94, 131.08, 128.96, 126.97, 125.91, 122.45, 122.42, 121.20, 120.09, 119.07, 113.83, 59.83, 58.20, 47.90, 45.54, 45.49, 45.32, 42.66, 36.01, 27.63, 24.46; HRMS (ESI): Calcd for [M + 2H]^2+^ (C_34_H_43_ClN_8_O_2_) requires m/z 316.1672, found 316.1683.

Oligomers/primers were purchased from Invitrogen (China). QPB-15e (10 mM) stock solutions were prepared using 10% dimethyl sulfoxide (DMSO) and stored at −70°C. Antibodies were purchased from Sangon, China. The two-step RT-PCR kit and the total RNA isolation kit were purchased from SBS Genetech, China. All tumor cell lines were obtained from the animal experimental center of Sun Yat-sen University.

This study was approved by the Biomedical Research Ethics Committee of Anhui Medical University. Animals were maintained and experiments were conducted at the laboratory animal center of Anhui Medical University. All procedures were in strict accordance with the guidelines for animal experiments set by the Biomedical Research Ethics Committee of Anhui Medical University.

### PCR stop assay

The PCR stop assay was performed using a modified protocol from the Master Cycler Personal (Eppendorf) [19]. The reaction mixture contained 2 μmol Pu27 oligomers (forward: 5 - TGGGGAGGGTGGG GAGGGTGGGGAAGG - 3; reverse: 5 - ATCGATCTCTT CTCGTCCTTCCCCA - 3), 2.5 U Taq polymerase, 0.16 μM dNTP, and a specified concentration of QPB-15e. PCR conditions were as follows: 94°C for 3 min, 10 cycles of 94°C for 30 s, 58°C for 30 s, and 72°C for 30 s. Amplified products were resolved with a 15% non-denaturing polyacrylamide gel in 1 TBE and silver staining.

### FRET–melting assay

FRET-melting was performed on a real-time PCR apparatus (Roche LigntCycler) as previously described [20]. Briefly, the fluorescent-labeled oligonucleotide FPu18T (5′ - FAM–AGGGTGGGGAGGGTGGGG–TAMRA - 3′) was denatured. Fluorescence measurements with the Pu18 oligonucleotide (0.2 μM) were taken in a buffer (pH 7.2) containing KCl. A mixture containing 10 μL of the compound solution (4 μM) and 10 μL FPu18T (400 nM) was annealed at 90°C for 5 min, then cooled to 37°C in a thermocycler. The solutions were added into LightCycler capillaries, and measurements were performed on the Roche LightCycler 2 with excitation at 470 nm and detection at 530 nm. The melting curves of the G-quadruplex DNA were obtained at 1°C intervals from 37°C to 99°C. The melting temperature Tm is the mid-point of a melting curve as measured by Origin 8.0 (OriginLab Corp.).

### CD measurement

CD measurements were performed on a Chirascan (Applied Photophysics) spectrophotometer [21]. The oligonucleotide Pu27 (5′ - TGGGGAGGGTGGGGAGGG TGGGGAAGG - 3′) at a final concentration of 5 μM was resuspended in Tris–HCl buffer (10 mM, pH 7.2) containing compound with or without KCl (100 mM) to be tested. The mixtures were heated to 95°C for 5 min, gradually cooled to 37°C, incubated at 4°C for 12 h, then recorded on the spectrophotometer using 0.5 s-per-point from 220 nm to 450 nm and 1 nm bandwidth. CD spectra were calculated as the average of two scans. Data analysis was carried out using Origin 8.0 (OriginLab Corp.).

### Competitive dialysis

All experiments were performed in a Tris-HCl buffer (10 mM, pH 7.2) with 100 mM NaCl. Dialysate solution (200 mL) containing QPB-15e (1 μM) was placed in a beaker for each competition dialysis assay [24]. Each nucleic acid sample (double strands, i-motif, single strands, triplet or quartet, a volume of 500 μL at 45 μM monomeric unit) was pipetted into a separate 0.5 mL DispoDialyzer (Spectrum Laboratories, Inc.). All 10 dialysis units were placed in a beaker, including the dialysate solution (10 mM Tris–HCl containing 1 μM, and stirred for 24 h at room temperature. Finally, all nucleic acid samples were carefully transferred to microcentrifuge tubes and treated with 1% sodium dodecyl sulfate (SDS). The compound concentration in each sample was measured by UV absorbance.

### Molecular modeling

The crystal structure of the parallel-type 18-mer *c-myc* G-quadruplex (Pu18, built by Tian–Miao Ou, *et al.*) [30] was used as the initial model for investigating the interaction between QPB-15e and *c-myc* DNA. The ligand was constructed in SYBYL 7.3.5 (Tripos Inc., St. Louis, MO, USA) and was charged using the Gasteiger–Huckel computational method. Docking studies were performed using the AUTODOCK 4.1 program. The dimensions of the active site box were set to 60Å × 60Å × 60Å with grid points 0.375Å apart to encompass the entire DNA molecule. Docking calculations were carried out using the Lamarckian genetic algorithm, with a population of random individuals (population size: 150), a maximum number of 25,000,000 energy evaluations, a maximum number of 27,000 generations, and a mutation rate of 0.02. Two hundred independent docking runs were performed for the ligand. All conformations of the ligand were chosen on the basis of the ligand's optimal binding arrangement with the G-tetrads and the lowest final docked energy.

### RT-PCR

After incubation with various concentrations of QPB-15e, cell pellets were dissolved in TRIzol solution. Total RNA was extracted in accordance with the manufacturer's instructions and mixed with distilled deionized water containing 0.1% diethyl pyrocarbonate (DEPC) to obtain a final volume of 50 μL. Each reaction mixture (20 μL) contained 1 × M-MLV buffer, 100 pmol oligo-dT primer, 100 U of M-MLV reverse transcriptase, 500 μM dNTP, 0.1% DEPC–H_2_O, and 1 mg of total RNA. The mixture was incubated at 42°C for 60 min for reverse transcription, and at 92°C for 10 min to inactivate the enzyme. PCR was carried out through the following steps. Each 20 μL reaction contained 1 × PCR buffer, 1.5 μM *c-myc* primers, 0.15 μM β-actin primers, 500 μM dNTPs, 0.1% DEPC–H_2_O, 1 U of Taq polymerase, and 3 μL of the cDNA template. Each reaction mixture was incubated in a thermal cycler as follows: 95°C for 5 min, 36 cycles of 95°C for 1 min, 50°C for 1 min, and 72°C for 1 min. The amplified products were separated in a 1.5% agarose gel, and images were obtained on a Gel Doc 2000 Imager System.

The primers used in the real-time RT-PCR were as follows: *c-myc*A, 5′ - TGGTGCTCCATGAGGAGACA - 3′; *c-myc*S, 5′ - GTGGCACCTCTTGAGGACCT - 3′; β-actinA, 5′ - GTTGCTATCCAGGCTGTGC - 3′, the upstream primer for β-actin; and β-actinS, 5′ - GCATCCTGTCG GCAATGC - 3′.

### Western blot

All cancer cells from each well of the culture plates were dissolved in 150 μL of extraction buffer, including 100 μL of solution A (25 mM Tris–HCl, pH 8, 50 mM glucose, 1 mM PMSF and 10 mM EDTA) and 50 μL of solution B (50 mM Tris–HCl, pH 6.8, 6% 2-mercaptoethanol, 6 M urea, 3% SDS, and 0.003% bromophenol blue). The mixture was centrifuged at 15,000 rpm and 4°C for 5 min, and the supernatant (10 μL for each sample) was loaded onto a 10% polyacrylamide gel and transferred onto a microporous polyvinylidene difluoride (PVDF) membrane. The experiment was performed using anti-β-actin or anti-*c-myc* primary antibody and horseradish peroxidase-conjugated anti-rabbit or anti-mouse secondary antibody. Finally, all protein bands were visualized using chemiluminescence substrates.

### Long-term cell culture experiments

Long-term proliferation experiments were performed using Ramos and CA46 lymphoma cell lines. Each cell line (1.0 × 10^5^) was grown in 10 cm Petri dishes and exposed to a subcytotoxic concentration of the ligand or an equivalent volume of 0.1% DMSO every 4 days. Cells in the control and drug-exposed dishes were counted and the dishes were each reseeded with 1.0 × 10^5^ cells. The remaining cells were collected and used for measurements as described below. This process was continued for 16 days.

### *In vivo* anti-tumor activity studies

All *in vivo* experiments were performed using 5-week-old female Balb/c-nude mice weighing 15–20 g. Mice were maintained in a laminar flow room with constant temperature and humidity. SMMC-7721 cells were inoculated subcutaneously into the dorsal sides of the mice (1 × 10^7^ cells/mice). After 7 days, when established tumors of 50 to 100 mm^3^ were detected, mice were treated with QPB-15e (10 or 20 mg/kg/day for 3 weeks). Control mice were given the vehicle only. Each control or drug-treated group included six mice bearing subcutaneous tumors. Tumors were implanted on day 0, and tumor diameters were measured daily for 3 days with a Vernier caliper. Tumor weight was calculated in accordance with the formula: Tumor volume (mm^3^) = d^2^ × D/2, where d and D are the shortest and the longest diameters, respectively. The inhibition ratio of the tumor was calculated with the formula: Inhibition ratio, IR (%) = 100% × (A − B)/A, where A and B are the average tumor weights of the control and the treated groups, respectively. *C-myc* mRNA and protein levels in tumor tissues were examined by RT-PCR and western blotting.
